# Quantitative Determination of Four Potential Genotoxic Impurities in the Active Pharmaceutical Ingredients in TSD-1 Using UPLC-MS/MS

**DOI:** 10.3390/molecules27134129

**Published:** 2022-06-27

**Authors:** Taiyu Wang, Hailong Yang, Jie Yang, Ningjie Guo, Guodong Wu, Xueyu Xu, Ming An

**Affiliations:** 1 Department of Pharmacy, Baotou Medical College, Inner Mongolia University of Science and Technology, Baotou 014060, China; taiyu_wang7@163.com (T.W.); wgdzd@126.com (G.W.); 2 Chemical Pharmaceutical Research Center, Tasly Academy, Tasly Holding Group Co., Ltd., Tianjin 300410, China; aileyang@163.com (H.Y.); gg6531676@163.com (J.Y.); 3 School of Pharmaceutical Sciences, Zhengzhou University, Zhengzhou 450001, China; ningjie_guo@163.com

**Keywords:** UPLC-MS/MS, genotoxic impurities, TSD-1, active pharmaceutical ingredients, quantitation, method validation

## Abstract

A novel method of ultra-performance liquid chromatography tandem mass spectrometry (UPLC-MS/MS) was developed for the identification and quantification of four potential genotoxic impurities (PGIs) in the active pharmaceutical ingredients of TSD-1, a novel P2Y_12_ receptor antagonist. Four PGIs were named, 4-nitrobenzenesulfonic acid, methyl 4-nitrobenzenesulfonate, ethyl 4-nitrobenzenesulfonate, and isopropyl 4-nitrobenzenesulfonate. Following the International Conference of Harmonization (ICH) guidelines, this methodology is capable of quantifying four PGIs at 15.0 ppm in samples of 0.5 mg/mL concentration. This validated approach presented very low limits (0.1512–0.3897 ng/mL), excellent linearity (coefficients > 0.9900), and a satisfactory recovery range (94.9–115.5%). The method was sufficient in terms of sensitivity, linearity, precision, accuracy, selectivity, and robustness and, thus, has high practicality in the pharmaceutical quality control of TSD-1.

## 1. Introduction

**Figure 1:**
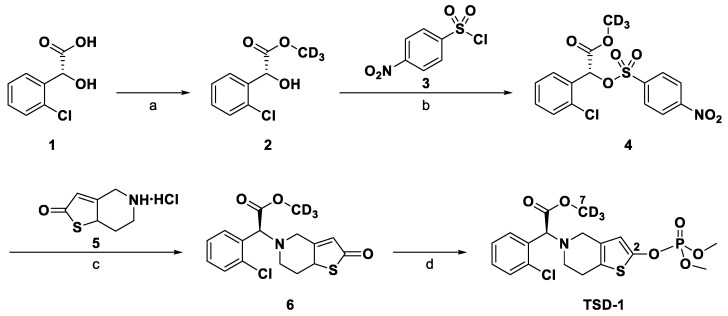
Synthetic route of TSD-1. Reagents and conditions: (a) methanol-*d*_4_, H_2_SO_4_, reflux; (b) DMAP, CH_2_Cl_2_, Et_3_N, −10 °C to −5 °C; (c) K_2_CO_3_, acetonitrile, r.t.; (d) dimethyl chlorophosphate, THF, DBU, −15 °C to −10 °C.

Clopidogrel is one of the most widely used antiplatelet drugs for the treatment of ischemic cerebrovascular disease and the secondary prevention of ischemic stroke [1,2,3]. However, approximately 16–65% of patients treated with clopidogrel show resistance due to the gene polymorphism, especially the CYP2C19 [3,4,5]. Furthermore, the majority (about 85%) of clopidogrel is hydrolyzed by carboxylesterase 1 (CES1) to inactive clopidogrel acid upon entry into the body [6,7]. Given the drawbacks of clopidogrel, a prodrug of deuterated clopidogrel active metabolites TSD-1, chemically named methyl-*d*_3_ 2-(2-chlorophenyl)-2-(2-((dimethoxyphosphoryl)oxy)-6,7-dihydrothieno[3,2-*c*]pyridin-5(4*H*)-yl)acetate, was researched and developed by Tasly Academy Chemical Pharmaceutical Research Center. Deuterium selectively replacing the methyl hydrogen at the C-7 reduces the methyl ester hydrolysis and enhances bioavailability. Meanwhile, dimethyl phosphate introduced at the C-2 of the thiophene ring minimizes the drug resistance caused by genetic polymorphism (Figure 1).

**Figure 2:**
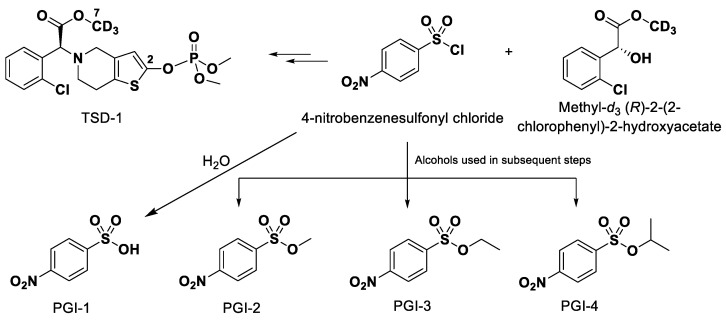
Formation of genotoxic impurities PGI-1, PGI-2, PGI-3, and PGI-4 during the synthesis of TSD-1.

The presence of potential genotoxic impurities (PGIs) in pharmaceutical substances and products continues to receive increased attention because of the related risks to human health. These PGIs can cause genetic mutations, chromosomal breakage, and chromosomal rearrangements and may cause cancer in human beings [8,9,10]. To ensure the safety of drug use, the European Drug Administration (EMA), the United States Food and Drug Administration (FDA), and the International Council of Harmonization (ICH) issued relevant guidelines on the control of PGIs in active pharmaceutical ingredients (APIs) [11,12,13]. The manufacture of APIs may produce PGIs; so, trace amounts of PGIs in drugs must be monitored and regulated to ensure public safety [14,15,16,17]. TSD-1 is synthesized from 4-nitrobenzenesulfonyl chloride. During the process of chemistry synthesis, residual 4-nitrobenzenesulfonyl chloride is hydrolyzed to form 4-nitrobenzenesulfonic acid (PGI-1) or react with the solvent used, including methanol, ethanol, and isopropanol to form methyl 4-nitrobenzenesulfonate (PGI-2), ethyl 4-nitrobenzenesulfonate (PGI-3), and isopropyl 4-nitrobenzenesulfonate (PGI-4), (Figure 2). All four compounds may remain in the API and be assessed as potentially genotoxic by (Q)SAR software (Toxtree Version 3.1.0, TOPKAT (BIOVIA toxicity prediction model) Version 6.2), since the threshold of toxicological concern (TTC) limit is 1.5 μg/day according to ICH M7 [18,19], and the limit is 15.0 ppm, when the daily dose of TSD-1 is 100 mg.

To date, some methods have been developed to determine sulfonate esters impurities, such as gas chromatography (GC) analysis [20], liquid chromatography tandem mass spectrometry (LC-MS) analysis [21], and gas chromatography tandem mass spectrometry (GC-MS) analysis [22]. To the best of our knowledge, no method has been systematically reported with multiple reaction monitoring (MRM) using ultra-performance liquid chromatography tandem mass spectrometry (UPLC-MS/MS) for detecting sulfonate. Moreover, no method has been reported for detecting PGI-1, PGI-2, PGI-3, and PGI-4 in the literature. In this study, a novel UPLC–MS/MS method with MRM mode was developed to investigate simultaneously four PGIs, which provide an essential basis for the production process and quality control of TSD-1.

## 2. Results and Discussion

### 2.1. Method Development

#### 2.1.1. Optimization of Sample Preparation

Sample preparation is an essential part of the PGI analysis, because matrix effects in trace analysis can be magnified, causing loss of sensitivity, abnormal recovery, and analyte instability. Different diluents were evaluated concerning the extraction efficiency and chromatography. The solubility of TSD-1 and the four PGIs was good in acetonitrile, isopropanol, and acetonitrile–water (50:50, *v*/*v*). Isopropanol and acetonitrile–water (50:50, *v*/*v*) were not suitable as diluents, due to the low responses and poor peak shape of the analytes. Acetonitrile alone was a good response, and proper peak shapes were obtained for TSD-1 and the four PGIs; in addition, consistent recoveries (91.5–107.1%) were obtained for all four PGIs using acetonitrile as diluent.

#### 2.1.2. Selection of Chromatographic Column and Separation

Three types of columns with different stationary phases, namely, Waters ACQUITY UPLC BEH C18 (2.1 mm × 100 mm, 1.7 µm), Waters ACQUITY UPLC BEH Amide (2.1 mm × 150 mm, 1.7 µm), and Waters ACQUITY UPLC HSS T3 C18 (2.1 mm × 100 mm, 1.8 µm), were evaluated in order to obtain proper separation of the four PGIs. Waters ACQUITY UPLC HSS T3 C18 (2.1 mm × 100 mm, 1.8 µm) provided better retention performance and improved peak shapes. Different compositions of mobile phase were investigated including water, 0.1% acetic acid (*v*/*v* in water), 0.1% aqueous ammonia (*v*/*v* in water), 5 mM ammonium acetate (*v*/*v* in water) with acetonitrile, including isocratic and gradient elution modes. Water and acetonitrile as the mobile phase with gradient elution mode achieved the optimum separation of the four PGIs. The flow rate of the mobile phase was 0.3 mL/min, and the column temperature was maintained at 30 °C. The auto-sampler temperature was held at 4 °C for sample stability. The retention times of approximately 0.79, 2.99, 3.24, and 3.53 min were observed for the four PGIs, respectively.

#### 2.1.3. Optimization of Mass Spectrometric Parameters

Choosing a detection method is an essential part of pharmaceutical analysis. Given this, the sensitive and specific mass spectrometric detection of LC-MS/MS was evaluated. Evaluation of the LC-MS/MS method was found to be unsuitable for the simultaneous detection and quantification of the PGIs in TSD-1 due to the poor resolution and unsatisfactory peak shapes. Furthermore, the possibility of electrospray ionization (ESI) source under positive and negative ion detection mode was evaluated during the early stage of method development. The signal intensity in negative mode was much higher than that in positive mode. Therefore, a selective and sensitive UPLC–MS/MS method with MRM mode was developed for the detection and quantification of the PGIs in TSD-1, and the electrospray ionization (ESI) source was operated in negative mode for ion detection. The appropriate response, satisfactory peak shapes, and accurate quantitation were obtained by optimizing the ion source parameters.

### 2.2. Method Validation

The developed UPLC-MS/MS method for the determination of the four PGIs in TSD-1 API was validated based on the ICH Q2 [23]. The methods were fully validated, including the specificity, limit of detection (LOD), limit of quantification (LOQ), linearity, accuracy, repeatability, intermediate precision, solution stability, and the robustness.

#### 2.2.1. Specificity

**Figure 3:**
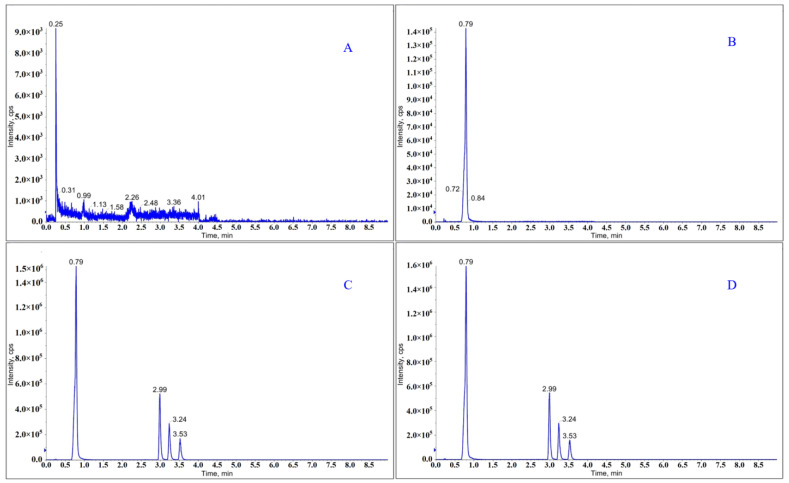
MRM chromatograms of (**A**) blank solutions, (**B**) TSD-1 sample solutions, (**C**) mixed standard solutions, and (**D**) sample spiked with mixed standard solutions.

No interference was observed at the retention times of PGI-1 (0.79 min), PGI-2 (2.99 min), PGI-3 (3.24 min), and PGI-4 (3.53 min) when blank solutions were analyzed (Figure 3).

#### 2.2.2. LOD, LOQ, and Linearity

**Figure 4:**
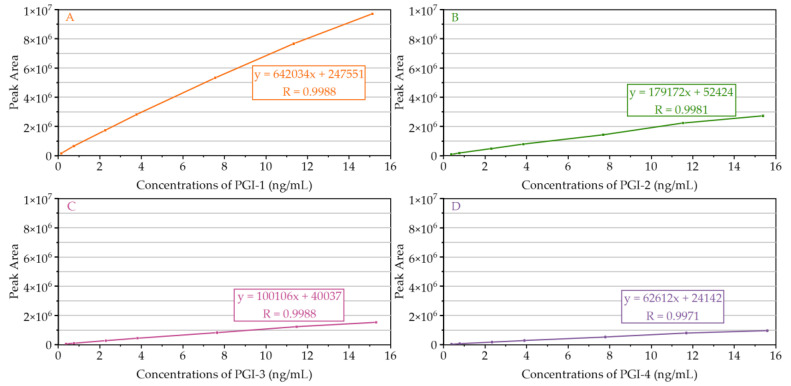
Calibration curves for the determination of (**A**) PGI-1, (**B**) PGI-2, (**C**) PGI-3, and (**D**) PGI-4 of seven levels of standard solutions.

The calibration curve was tested by running seven levels of standard solutions, and the linear regression analysis of the peak area ratios versus nominal concentrations was fitted over these concentration ranges (Figure 4). The linear range, regression equation, correlation coefficient, LOD, and LOQ of the four PGIs are shown in Table 1. All calibration curves exhibited good linearity, with correlation coefficients greater than 0.9900. The LOQ of PGI-1, PGI-2, PGI-3, and PGI-4 were 0.1512 ng/mL, 0.3844 ng/mL, 0.3826 ng/mL, and 0.3897 ng/mL, respectively. The S/N for LOD solutions were between 7 and 21 and for LOQ solutions were between 19 and 63 in the case of each PGI, which indicated that the method was considered satisfactory and adequate for the four PGIs.

#### 2.2.3. Accuracy

The method accuracy was evaluated as the recovery of the spiked samples of the tested substance, at concentrations at LOQ, 100%, and 150% concentration levels, respectively. The mean recovery was between 94.9% and 115.5% (Table 2). The relative standard deviation (RSD) was below 5.0%. These results demonstrate the accuracy of the developed method.

#### 2.2.4. Repeatability and Intermediate Precision

The mean recovery results were calculated from the experimental data of both analysts between 92.1% and 111.4%. Moreover, the RSD results were less than 4.0% for each PGI. The experimental data are presented in Table 3. These results prove the repeatability and intermediate precision of the method.

#### 2.2.5. Solution Stability

The solution stability was carried out using mixed standard solutions and spiked sample solutions in an auto-sampler. The peak areas of all the four PGIs were determined at 1.5–10.0 h intervals for the study period of 34 h. There were no significant changes observed for all the four PGIs. The RSD of the peak areas (*n* = 12) were 6.4%, 2.1%, 5.8%, and 6.4% for all the four PGIs in the standard solutions; the RSD of the peak areas (*n* = 12) were 3.4%, 4.7%, 6.1%, and 6.9% for all the four PGIs in the spiked sample solutions. Therefore, we confirmed the stability of the PGIs in the sample solutions for at least 34 h.

#### 2.2.6. Robustness

The robustness of our method was tested by altering the flow rate and the column temperature as well as the proportion of the mobile phase. The recovery of the spiked sample solutions was calculated to evaluate the robustness of the method. The results showed no considerable change in chromatographic performance of all four PGIs in the mentioned changes, as presented in Table 4. The validated UPLC–MS/MS method was considered robust since the RSDs for the PGI-1, PGI-2, PGI-3, and PGI-4 were 0.5%, 10.6%, 4.6%, and 4.7%, respectively, across all conditions.

### 2.3. Analysis of TSD-1 API Batches

To potentially detect the aforementioned PGIs, samples from three API batches (Lot No. 181104, 181105, 181106) were analyzed using our validated UPLC-MS/MS method. The test concentration of TSD-1 was 0.5 mg/mL. The results showed that for PGI-1, except for batch 181106 (17.4 ppm, RSD = 1.4%), the batches were less than 0.3 ppm, which was below the range of LOQ detection; PGI-2, PGI-3, and PGI-4 were not detected in the three TSD-1 batches, which indicated that two batches met the requirement of the total PGIs limit (15.0 ppm).

## 3. Materials and Methods

### 3.1. Chemicals and Reagents

TSD-1 API (Lot No. 181104, 181105, 181106) was manufactured at the Tasly Academy Chemical Pharmaceutical Research Center. Acetonitrile LC-MS grade (Merck, Darmstadt, Germany), Reference substances of PGI-1 (purity: 98.21%) and PGI-2 (purity: 99.34%) were purchased from Aladdin (Shanghai, China). Reference substances of PGI-3 (purity: 99.51%) and PGI-4 (purity: 99.15%) were synthesized at the Tasly Academy Chemical Pharmaceutical Research Center. High pure Milli-Q water was used with the help of a Millipore Milli-Q plus purification system (Millipore, Billerica, MA, USA).

### 3.2. Solutions for Method Validation

We accurately weighed 15 mg of PGI-1, PGI-2, PGI-3, and PGI-4, respectively, into a 100 mL volumetric flask, diluted to volume with acetonitrile, and shook well; we transferred 1 mL, accurately measured, to a 200 mL volumetric flask, diluted to volume with acetonitrile, shook well, and used as the mixed standard stock solutions (containing 750.0 ng/mL of the four PGIs). We transferred 1 mL of the mixed standard stock solutions to a 100 mL volumetric flask, diluted to volume with acetonitrile, shook well, and used as the mixed standard solutions (containing 7.5 ng/mL of the four PGIs). For linearity validation, the mixed standard stock solutions were diluted using acetonitrile to give standards at 0.75, 2.25, 3.75, 7.50, 11.25, and 15.00 ng/mL (1.5, 4.5, 7.5, 15.0, 22.5, and 30.0 ppm, respectively). We accurately weighed 25 mg of TSD-1 to a 50 mL volumetric flask, diluted to volume with acetonitrile, shook well, and used as the TSD-1 sample solutions (containing 0.5 mg/mL of TSD-1). The spiked sample solutions were prepared by accurately weighing 25 mg of TSD-1 in 50 mL volumetric flask further dissolved and diluted in the mixed standard solutions. To perform the recovery experiment, the spiked recovery solutions were prepared by accurately weighing 25 mg of TSD-1 in a 50 mL volumetric flask further dissolved and diluted in mixed standard solutions at the limit of quantification concentration, 100% limit level concentration, and 150% limit level concentration, respectively.

### 3.3. Chromatographic and Mass Spectrometric Conditions

The UPLC-MS/MS method was developed using a UPLC system (Shimadzu, Kyoto, Japan) consisting of a quaternary pump (LC-30AD), an auto-sampler (SIL-30AC), and a column oven (CTO-30A), in combination with a QTrap 5500 triple quadrupole MS detector (AB Sciex, Foster City, CA, USA). Prior to injection in the MS system, the samples were eluted on a Waters ACQUITY UPLC HSS T3 C18 (2.1 mm × 100 mm, 1.8 µm) (Waters, Milford, MA, USA).

The mobile phase A was high pure Milli-Q water, and mobile phase B was acetonitrile. The gradient elution program was as follows: 0–0.5 min, 20% B; 0.5–1.5 min, 20% B to 65% B; 1.5–4.0 min, 65% B; 4.0–6.0 min, 65% B to 20% B; and 6.0–9.0 min, 20% B. In order to avoid any pollution due to the high TSD-1 concentrations over the mass spectrometry (MS) procedures, a switch valve was fitted between the UV and MS detectors. MS acquisition time was set to 0–4.0 min. After 4.0 min, the sample entered the waste liquid. The flow rate and the injection volume were 0.3 mL/min and 2.0 µL. The column temperature was maintained at 30 ◦C, while the auto-sampler temperature was maintained at 4 °C. The MRM mode for the mass spectrometer, using a negative ion mode electrospray ionization (ESI), was applied to monitor the PGIs. The quantitative ion pairs were *m*/*z* 202→138 and simultaneous quantification of four PGIs. The electrospray source parameters were fixed as follows: analyses were performed with ion spray voltage −4500 V; ion source temperature 550 °C; nebulizer gas (GS1) 45 psi; nebulizer gas (GS2) 45 psi; collisionally activated dissociation (CAD) medium; curtain gas (CUR) 35 psi; declustering potential (DP) −100 V; collision energy (CE) −25 V; and cell exit potential (CXP) −10 V and entrance potential (EP) −10 V.

### 3.4. Method Validation

The specificity of the method was determined by comparing the chromatograms of the blank solutions, sample solutions, standard solutions, and the spiked sample solutions. LOD and LOQ for the four PGIs were validated based on signal-to-noise (S/N) ratios of ≥3 and ≥10 by injecting each known standard concentration six times. The linearity of the evaluated method was demonstrated with seven concentration levels in the range of LOQ to 200% of the permitted level of PGIs using the least square regression method. Standard addition and recovery experiments were conducted for the pure samples to determine the accuracy of the method. The method’s accuracy was evaluated in triplicate using three concentration levels at LOQ, 100%, and 150% concentration levels, respectively. Then the recovery and the RSD for the four PGIs were calculated. The repeatability assessed by quantifying the spiked sample solutions was carried out to assess the method’s ability to quantify samples by injecting six replicate injections of the same analyst. For intermediate precision, the same solutions were used, and another analyst performed the analysis. The solution stability was determined by analyzing the standard solutions and spiked sample solutions, keeping them in an auto-sampler, and observing the variations in their peak areas. The standard solutions were re-analyzed at different time intervals, and we calculated the RSD of the peak area. The robustness of the method was analyzed with deliberate alteration in the flow rate of the mobile phase, the column temperature, and the proportion of mobile phase. The optimized flow rate of the mobile phase was 0.30 mL/min, and the same was altered by ±0.02 units, i.e., from 0.28 mL/min to 0.32 mL/min. The effect of the column oven temperature into resolution was studied at 28 ◦C and 32 °C (instead of the optimized temperature, 30 °C). The percentage of acetonitrile was 20%, and the performance of the method was studied at 18 and 22%.

## 4. Conclusions

The formation of genotoxic impurities in the synthesis of TSD-1 should be avoided, whether as a single genotoxic impurity or multiple genotoxic impurities. If removing genotoxic impurities is not technically feasible, then an accurate detection method must be determined for potential genotoxic impurities. The aim of this study was to develop a sensitive analytical method, which was able to determine the traces of four sulfonate genotoxic impurities in TSD-1. Therefore, an original UPLC-MS/MS method was developed to quantitatively determine these PGIs at targeted levels and evaluated. The method was completely validated and showed good specificity, LOD, LOQ, linearity, accuracy, repeatability, intermediate precision, solution stability, and robustness. The LOQ values for the PGIs were well below the allowed limits. In addition, the methodology was successfully applied to PGIs in bulk batches of TSD-1, reiterating its high efficacy at low levels. Therefore, this method may serve as a rapid, precise, and accurate platform to quantify PGI-1, PGI-2, PGI-3, and PGI-4 in TSD-1 during quality assurance tests.

## Figures and Tables

**Table 1:** LOD, LOQ, and linear regression equation of PGI-1, PGI-2, PGI-3, and PGI-4.

Compound	Linear Range (ng/mL)	Calibration Curve Equation	Correlation Coefficient (R)	LOD (ng/mL) ± RSD (%)	LOQ (ng/mL) ± RSD (%)
PGI-1	0.1512–15.1158	*y* = 642034*x* + 247551	0.9988	0.0453 ± 1.9	0.1512 ± 1.1
PGI-2	0.3844–15.3778	*y* = 179172*x* + 52424	0.9981	0.1153 ± 2.5	0.3844 ± 1.4
PGI-3	0.3826–15.3046	*y* = 100106*x* + 40037	0.9988	0.1148 ± 0.9	0.3826 ± 1.5
PGI-4	0.3897–15.5864	*y* = 62612*x* + 24142	0.9971	0.1169 ± 1.4	0.3897 ± 2.8

**Table 2:** Summary of accuracy results (*n* = 3).

Spike Level		PGI-1	PGI-2	PGI-3	PGI-4
LOQ	Mean ± SD (%)	94.9 ± 3.4	106.2 ± 4.7	111.4 ± 4.1	115.5 ± 2.8
	RSD (%)	3.6	4.4	3.7	2.4
100%	Mean ± SD (%)	101.3 ± 0.5	102.0 ± 1.4	100.0 ± 1.0	102.1 ± 0.6
	RSD (%)	0.5	1.4	1.0	0.6
150%	Mean ± SD (%)	98.6 ± 0.7	100.3 ± 1.9	100.3 ± 3.2	100.9 ± 0.5
	RSD (%)	0.7	1.9	3.2	0.5

**Table 3:** Repeatability and intermediate precision for the developed UPLC-MS/MS method (*n* = 6).

Compound		PGI-1	PGI-2	PGI-3	PGI-4
Analyst 1	Mean ± SD (%)	97.7 ± 1.5	111.4 ± 1.0	92.1 ± 1.1	97.5 ± 2.0
	RSD (%)	1.5	0.9	1.2	2.1
Analyst 2	Mean ± SD (%)	98.6 ± 2.1	101.2 ± 3.4	97.8 ± 2.0	102.9 ± 3.3
	RSD (%)	2.1	3.3	2.1	3.2

**Table 4:** Robustness study for the recovery of the four PGIs.

Compound	Original Condition	Flow Rate (mL/min)		Column Temperature (°C)		Percentage of Acetonitrile (%)		Mean ± SD (%)	RSD (%)
		0.28	0.32	28	32	18	22		
PGI-1	99.7	99.9	99.5	99.3	99.4	98.5	99.6	99.4 ± 0.5	0.5
PGI-2	112.5	107.1	123.0	127.6	112.5	97.8	96.2	111.0 ± 11.8	10.6
PGI-3	90.4	94.7	102.7	101.7	94.1	96.4	99.1	97.0 ± 4.4	4.6
PGI-4	94.3	106.2	99.8	99.5	106.4	107.6	102.0	102.3 ± 4.8	4.7

## Data Availability

Not applicable.
